# Multi-parametric MRI as an indirect evaluation tool of the mechanical properties of *in-vitro* cardiac tissues

**DOI:** 10.1186/1471-2261-13-24

**Published:** 2013-03-27

**Authors:** Delphine Périé, Nagib Dahdah, Anthony Foudis, Daniel Curnier

**Affiliations:** 1 École Polytechnique, Mechanical Engineering, Montréal, QC, Canada; 2 Research center, CHU Sainte-Justine, Université de Montréal, Montréal, QC, Canada; 3 Université de Montréal, Kinesiology, Montréal, QC, Canada

**Keywords:** Cardiac muscle, Myocardium, Ventricle, Mechanical properties, Multi-parametric MRI, Multiple regressions, Principal component analysis

## Abstract

**Background:**

Early detection of heart failure is essential to effectively reduce related mortality. The quantification of the mechanical properties of the myocardium, a primordial indicator of the viability of the cardiac tissue, is a key element in patient’s care. Despite an incremental utilization of multi-parametric magnetic resonance imaging (MRI) for cardiac tissue characteristics and function, the link between multi-parametric MRI and the mechanical properties of the heart has not been established. We sought to determine the parametric relationship between the myocardial mechanical properties and the MR parameters. The specific aim was to develop a reproducible evaluative quantitative tool of the mechanical properties of cardiac tissue using multi-parametric MRI associated to principal component analysis.

**Methods:**

Samples from porcine hearts were submitted to a multi-parametric MRI acquisition followed by a uniaxial tensile test. Multi linear regressions were performed between dependent (Young’s modulus E) and independent (relaxation times T1, T2 and T2*, magnetization transfer ratio MTR, apparent diffusion coefficient ADC and fractional anisotropy FA) variables. A principal component analysis was used to convert the set of possibly correlated variables into a set of linearly uncorrelated variables.

**Results:**

Values of 46.1±12.7 MPa for E, 729±21 ms for T1, 61±6 ms for T2, 26±7 for T2*, 35±5% for MTRx100, 33.8±4.7 for FAx10^-2^, and 5.85±0.21 mm^2^/s for ADCx10^-4^ were measured. Multi linear regressions showed that only 45% of E can be explained by the MRI parameters. The principal component analysis reduced our seven variables to two principal components with a cumulative variability of 63%, which increased to 80% when considering the third principal component.

**Conclusions:**

The proposed multi-parametric MRI protocol associated to principal component analysis is a promising tool for the evaluation of mechanical properties within the left ventricle in the *in vitro* porcine model. Our *in vitro* experiments will now allow us focused *in vivo* testing on healthy and infracted hearts in order to determine useful quantitative MR-based biomarkers.

## Background

Heart failure is a progressive disease in which the damage to the cardiac tissue can be of primary or secondary origin. It entails incapacity of the myocardium to sustain an adequate blood flow for the systemic needs of the organism. It is a major health problem approaching epidemical proportions in industrialized countries and imputing billions of dollars in the healthcare resources. It is estimated that at least one third of adults over 55 years old will develop heart failure later in life. The ultimate risk of heart failure is accrued death, with a survival rate of 35% five years after diagnosis [1]. Echocardiography is a cornerstone in establishing the diagnosis of heart failure by measuring parameters expressing the physical changes attributable to the end result of physiopathological abnormalities. Whereas systolic dysfunction is easily and reliably quantified my measuring ejection and shortening fractions, myocardial disturbances preceding low cardiac output remain difficult to assess and largely operator dependent in daily clinical practice despite significant advances in quantitative echocardiography. We believe that the evaluation of the mechanical parameters of the myocardium would allow an early diagnosis of biomechanical myocardial changes leading to clinically relevant myocardial dysfunction before the onset of functional incapacity.

The mechanical properties of the myocardium are a primordial indicator of the viability of the cardiac tissue and heart failure. Uniaxial, biaxial and equibiaxial stretching tests were performed on excised ventricular samples. However, translating knowledge from freshly euthanized animals to live functioning hearts with reliable measures of the mechanical properties of the myocardium remains difficult because of the vascularity of the tissue that changes drastically immediately after death. Moreover, these mechanical properties vary according to the experimental loading protocol and the mathematical model. To solve the finite elasticity stress estimation problem, finite element models of the myocardium were constructed, from isotropic, initially spherical, membrane models [2] to realistic models based on multiple short-axis and long-axis MRI slices [3-6]. Cine-tagging MRI allowed the computation of the strain field within the myocardium on the basis of tags displacement within the heart over the cardiac cycle, and associated to the finite element models, the computation of the stress field and the material parameters [7-10]. With muscle fibre architecture from diffusion tensor imaging and deformation from tissue cine-tagging MRI included to the finite element models, the constitutive parameters of a hyperelastic transversely isotropic material law were determined by minimizing the difference between the predicted and imaged deformation field [11-15].

However, the mechanical properties depend on the finite element model and its validation. Thus, techniques allowing the direct measure of these properties from medical imaging were introduced. Magnetic resonance elastography was proposed with specific gradient-echo sequences to reach small echo times and low excitation frequencies adapted for the myocardium [16-20]. However, this technique requires a special system to create the vibrations and customized phase-contrast sequences to perform motion sensitization and remains a research application. Cine-DENSE sequence that encodes tissue displacement directly into the image phase was introduced to quantify myocardial strains [21,22]. However, this sequence is time consuming and requires previously segmented data prior to calculate the Lagrangian displacement field [23,24]. Associated to MRI phase contrast velocity mapping that allows the quantification of the relative pressure distribution from the Navier–Stokes equation, cine-DENSE MRI can be used to estimate the myocardial elastic modulus and viscosity from the strain-pressure relationship [22].

There is an incremental utilization of multi-parametric magnetic resonance imaging (MRI) for cardiac tissue characteristics and function. Maps of the longitudinal relaxation time T1 of the myocardium after injection of a contrast product allowed quantifying changes in heart failure models, reflecting tissue fibrosis [25]. Higher T1 values within the myocardium were found to be associated with reduced systolic function [26]. The maps of the transverse relaxation time T2 allowed an accurate detection of myocardic oedema [27] and were found to be associated with changes in water content within the tissue during ischemia [28]. The T2 fluctuations may reflect hyperemia and tissue cellular edema in accord with the known pathophysiology of ischemic and post-ischemic yet viable muscle [29]. Myocardial fibrosis and blood oxygenation did not seem to influence T2* [30]. The diffusion anisotropy was used to obtain information on the spatial architecture of the musculoskeletal or cardiac muscle, especially the collagen fibber tractography [31-37]. The disorganized fibbers were found to be consistent with the infracted regions and correlated with the alteration of the mechanical and histological properties of the tissue. In an animal model, a magnetization transfer acquisition with injection of gadolinium allowed the differentiation between infracted regions, regions of incapacity, and normal regions of the myocardium [38]. However, the link between multi-parametric MRI and the mechanical properties of the heart has not been established. We hypothesized that a relationship exists between the mechanical properties and the MR parameters of cardiac tissue. The specific aim of this study was to develop an indirect evaluation tool of the mechanical properties of cardiac tissue using multi-parametric MRI and principal component analysis.

## Methods

### Samples preparation

Porcine hearts (n=12) were obtained from a local slaughterhouse (Lavallée, Havelock, QC, Canada) within 2 hours of death. A square sample of 10 cm*6 cm*4 cm was dissected from the left ventricular myocardial tissue of each isolated heart and placed in a chamber filled with a tyrode saline solution (8 g of NaCl, 0.199 g of KCl, 0.204 g of CaCl2, 0.098 g of MgCl2, 1.0 g of NaHCO3, 0.053 g of NaH2PO3, and 0.998 g of dextrose within 1 l of water) at room temperature. Each sample was submitted to a multi-parametric MRI acquisition 4 to 6 hours after the tissue preparation, followed by a uniaxial tensile test one hour after the MRI acquisition.

### Multi-parametric MR imaging

The chamber was placed within the head coil of a 3 Teslas whole body system (Philips Achieva X-Series) and a single slice, 5 mm thick, was taken centered within the tissue sample. Images for the quantification of T1 and T2 were acquired using a multiple inversion recovery turbo spin-echo sequence for T1 (repetition time of 2100 ms, echo time of 6.3 ms, 15 inversions times from 50 to 1900 ms) and a multi-echo turbo spin-echo sequence for T2 (repetition time of 2000 ms, 10 echo times every 15 ms). T1 [39] and T2 were extracted (Matlab, r2007 Mathworks, Natick, MA) from the signal intensity using exponential relationships. The magnetisation transfer ratio MTR was obtained using two gradient echo sequences (repetition time of 83 ms, echo time of 3.8 ms), one with an off-resonance pulse applied at 1100Hz down to the free water proton resonance frequency and the other one without it [40]. MTR was calculated as described previously [41]. The last sequence measured the apparent diffusion coefficient ADC and the fractional anisotropy FA using a multi-shot spin-echo echo-planar-imaging diffusion-weighted sequence (repetition time of 2000 ms, echo time of 40 ms) with 15 non-collinear diffusion and a b value of 1000 s/mm^2^. ADC and FA were calculated as described previously [42-44].

**Figure 1:**
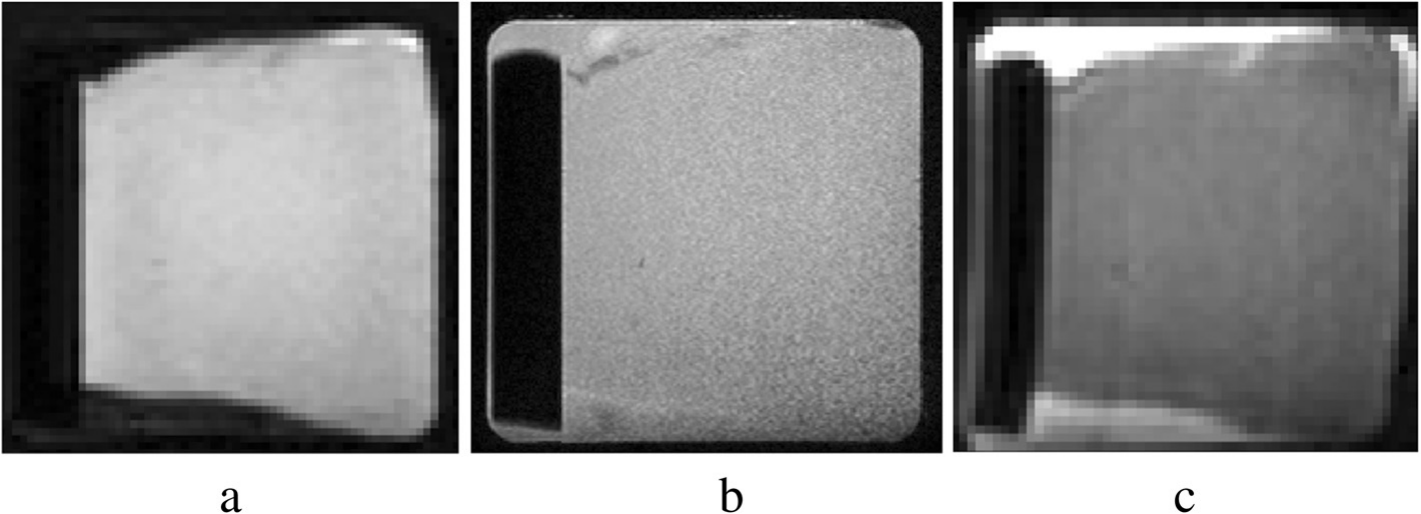
**T1-weighted (a), T2-weighted (b) and diffusion weighted with a b-value of 1000 s/mm**^**2 **^**(c) images of one cardiac tissue sample installed in the chamber manufactured in acrylonitrile butadiene styrene by fused plastic deposit.**

The mean and standard deviation of T1, T2, MTR, FA and ADC were calculated over a square region of interest (ROI) chosen in the middle of the tissue. The sensitivity of the determination of the mean MRI parameters over the ROI to the ROI location within the slice was very low, due to a spatially uniform signal, whatever the image weighting (Figure 1).

### Mechanical testing

**Figure 2:**
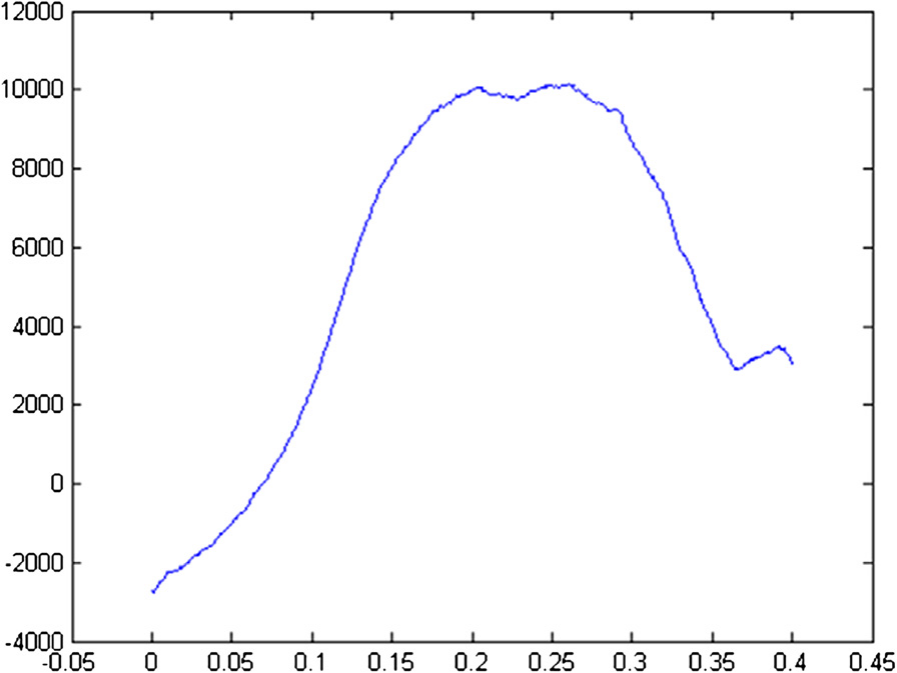
**Representative pressure (Pa) - stretch curve for all the samples obtained from the traction test until failure.** The resulting curve is composed of a non-linear toe region (stretch between 0 and 0.1), a linear region (stretch between 0.1 and 0.15), a plastic region (stretch between 0.15 and 0.3) and a failure region (stretch over 0.3). The Young’s modulus E is the slope of the curve in the linear part.

Immediately after the MRI acquisition, the heart tissue from the left ventricle was cut into samples of 5 cm*1 cm*1 cm, which were submitted to a preloading of 2 N followed by a ramp-release preconditioning of amplitude 3 mm and constant speed of 5 mm/s to align the fibres for 5 minutes according to previous protocols [45,46]. Then each sample was submitted to a uniaxial tensile test until failure with a constant speed of 1 mm/s (micro-mechanical testing system Mach-1, Biomomentum Inc.). The Young’s modulus E is the slope of the stress–strain curve in the linear part (Figure 2).

### Relationships between mechanical properties and MRI parameters

Multi linear regressions were performed between dependent (E) and independent (T1, T2, MTR, FA and ADC) variables to verify our hypothesis. In order to account for potential interaction between MR parameters, a principal component analysis was used to convert the set of possibly correlated variables into a set of linearly uncorrelated variables. The data were rendered to Z-values by subtracting the individual results from the group’s average, and dividing by the calculated group’s standard deviation. The covariance matrix and its eigenvectors and eigenvalues were computed. The cumulative energy content for each eigenvector was used to select a subset of eigenvectors as basis vectors. The source data were then converted into the new basis. The first principal component (F1) has the largest possible variance, and each succeeding component (F2, F3, …Fn) in turn has the highest variance possible under the constraint that it is orthogonal to the preceding components. All statistical tests were performed using XLSTATS (Addinsoft, New York, United States). All results were expressed as Mean±SD and the significance of all tests was set to p≤0.05.

## Results

**Figure 3:**
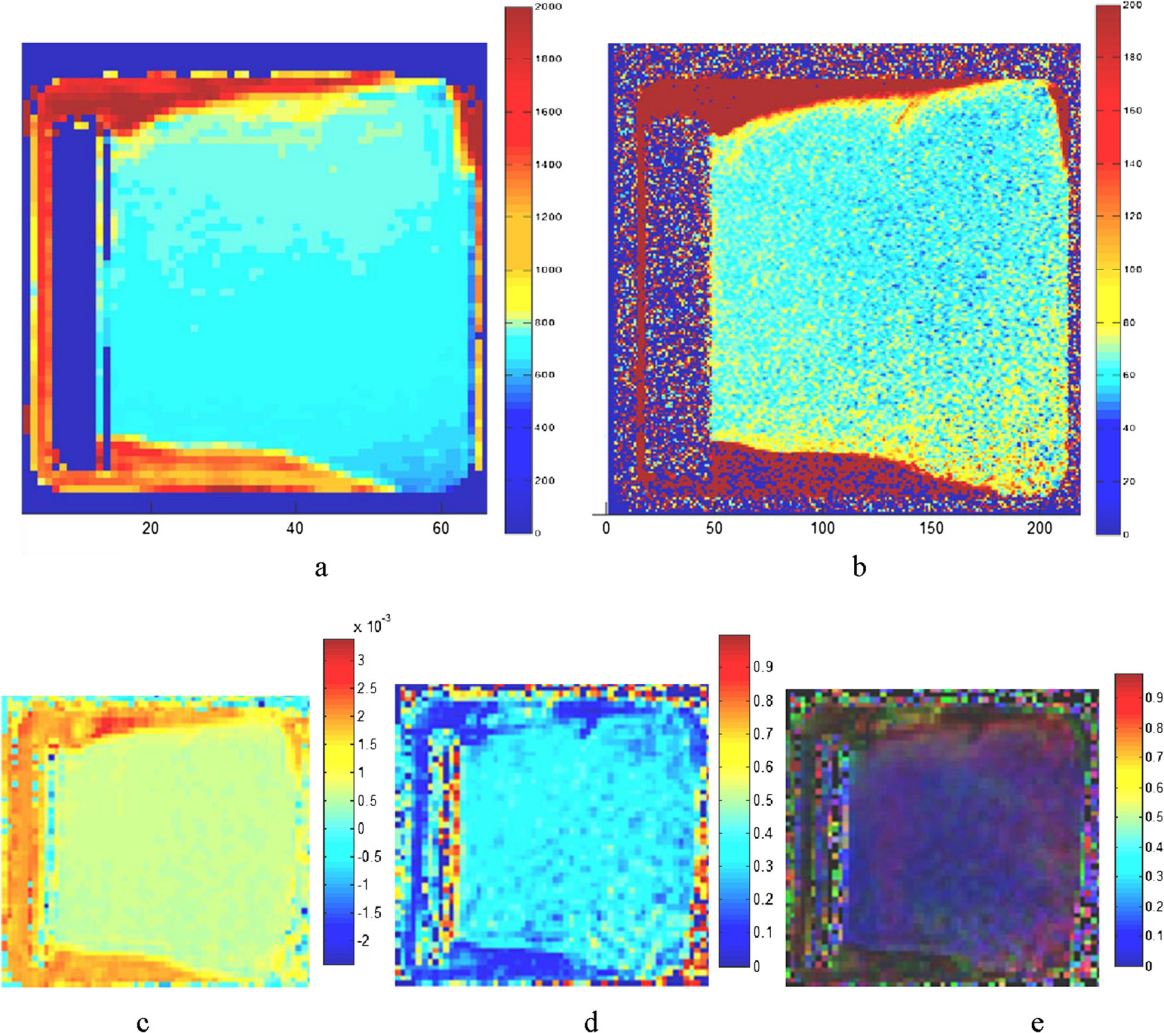
**T1 in ms (a), T2 in ms (b), ADC in mm**^**2**^**/s (c), FA (d) and FA color (e) maps calculated from the T1- and T2-weighted images and from the diffusion tensor images of the sample shown in Figure**1**.** For the FA color map, the colors indicate direction of fiber tracts (red transverse, blue cranio-caudal, green anterior–posterior).

All parameters measured (Table 1) showed a low standard deviation, with a normal distribution according to p-values over 0.05 for Shapiro-Wilk normality tests. The maps of the relaxation times and diffusion parameters (Figure 3) were uniform through the entire tissue sample.

**Table 1:** Mean and standard deviations of the twelve samples on the Young’s modulus E and the MRI parameters (T1, T2, T2*, MTR, FA and ADC)

***Property***	***Mean±SD***
E	46.1±12.7 MPa
T1	729±21 ms
T2	61±6 ms
T2*	26±7
MTRx100	35±5%
FAx10^-2^	33.8±4.7
ADCx10^-4^	5.85±0.21 mm^2^/s

Multi linear regressions showed that only 45% of E can be explained by the MRI parameters T1, T2, T2*, MTR, FA and ADC (Equation 1). The highest variance inflation factor was attributed to T2 when the regression was done on the 6 MRI parameters and to T2* when the regression was done on the remaining 5 MRI parameters (without T2). The multiple linear regression done after removing T2 and T2* (Equation 2) showed that all parameters had small and equivalent variance inflation factor and that the coefficient of determination did not change significantly (42%).

(1) $$\begin{array}{l} E=354911-419*T1-90*T2-499*T2*\\ \;-639*\text{MTR}-46150*\text{FA}+91555791*\text{ADC} \end{array}$$

(2) $$\begin{array}{l} E=329277-415*T1-210*\text{MTR}-882*\text{FA}\\ \;+46622057*\text{ADC} \end{array}$$

**Figure 4:**
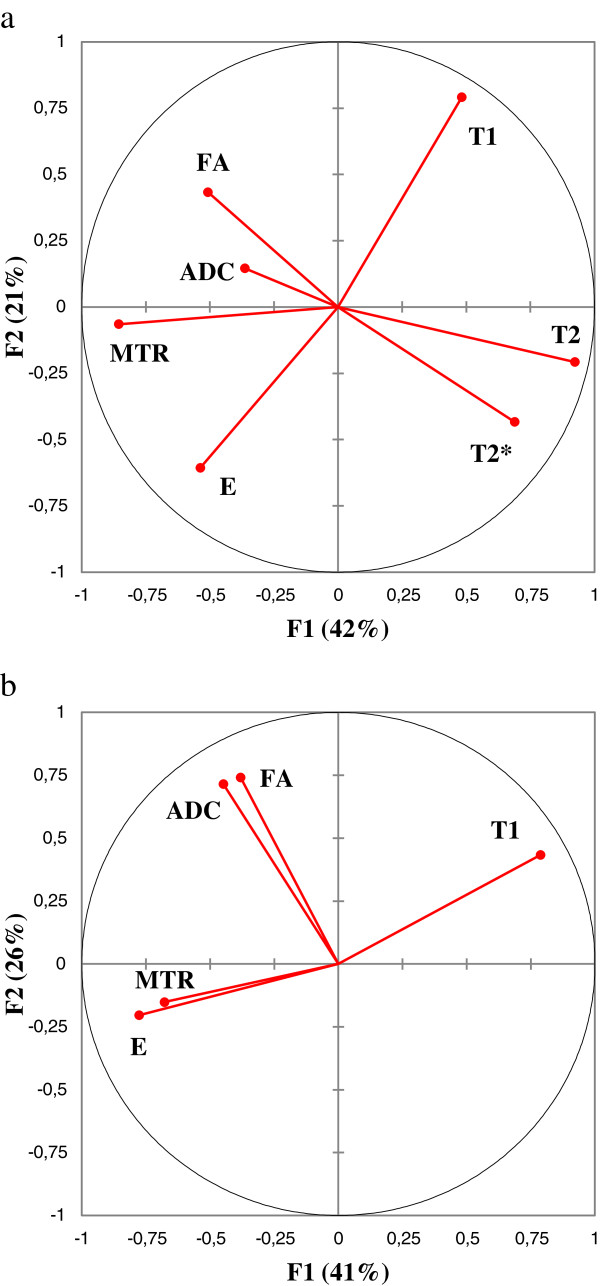
Correlation circle representing a) the 7 variables (E, T1, T2, T2*, MTR, ADC, FA) and b) the 4 variables (E, T1, ADC, FA) in the plane of the principal components (F1, F2).

The principal component analysis reduced our 7 variables (E, T1, T2, T2*, MTR, ADC, FA) to two principal components F1 and F2 with a cumulative variability of 63%, which increased to 80% when considering the third principal component F3. The representation of the 7 variables in the (F1, F2) plane (Figure 4-a) showed negative correlations between T1 and E as they were located near the circle and symmetric relatively to the circle origin. The coordinate of each variable in the (F1, F2) plane corresponds to the angle cosine (or correlation coefficient) between the variable and the axis, and the circle represents the sum of square cosines equal to 1. The position of T2 and MTR near the circle and the X-axis suggested that these parameters were expressed mainly by F1. The position of T1, T2* and E near the circle suggested that these parameters were expressed mainly by F1 and F2 (Equation 3). ADC and FA were far away from the circle, which suggested that these parameters were not expressed only by F1 or F2. The eigenvectors of the covariance matrix showed that they were expressed mainly by F3.

(3) $$E_{\text{centered}-\text{reduced}}=0.29*F1+0.37*F2$$

The principal component analysis reduced the 5 variables (E, T1, MTR, ADC, FA) to two principal components F1 and F2 with a cumulative variability of 67%, which increased to 81% when considering the third principal component F3. The representation of the 5 variables in the (F1, F2) plane (Figure 4-b) showed a positive correlation between ADC and FA. The position of T1, FA, and ADC near the circle suggested that these parameters were expressed mainly by F1 and F2. The position of E and MTR far away from the circle suggested that these parameters were not expressed only by F1 or F2. The eigenvectors of the covariance matrix showed that they were expressed mainly by F3 (Equation 4).

(4) $$E_{\text{centered}-\text{reduced}}=0.60*F1+0.04*F2+0.24*F3$$

## Discussion

We confirmed our hypothesis that a relationship exists between the Young’s modulus and the MRI parameters of the left ventricular myocardial tissue, and that this relationship may be in part non linear. Principal component analysis is very useful to reduce the dimensionality of a data set by projecting high dimensional data into a lower dimensional space. For the first time to our knowledge, a multi-parametric MRI acquisition composed of relaxation times mapping, magnetisation transfer and diffusion tensor imaging followed by a mechanical test in traction were performed on fresh porcine cadaveric hearts.

Hyperelastic properties or coefficients of the exponential stress-stretch relation were reported from bi-axial traction tests performed on bovine heart samples, but the stress-stretch curves showed various Young’s moduli from 2-7 kPa [46-49] to 150 kPa [45,50]. An elastic modulus of 30 kPa was reported for muscle strips of rats left ventricular walls tested in biaxial traction [51]. The stiffness modulus of human samples increased from 100 to 300 kPa with increasing collagen content [52]. There is a large variation between the reported moduli, but they are smaller than the ones we measured (46 MPa) on samples of porcine left ventricular walls, because of the different mechanical protocols in uniaxial or biaxial traction. Along-fibre moduli of 4-100 kPa were reported from finite element modeling [6]. Effective shear stiffness of 10-15kPa was reported from magnetic resonance elastography performed in vivo on pigs [20]. However, much higher stiffness coefficients were found in the fibre direction (2.6GPa) from ultrasonic velocity measurements on freshly excised ovine hearts [53].

The relaxation times we measured in this study on the isolated porcine left ventricular wall tissue were in the same range as the ones reported in the literature on animals or humans. Relaxography of excised rat myocardium showed T1 values of 907±77 ms, T2 values of 32±6 ms and T2* values of 32±6 ms [28]. T2 values of 50-60 ms [54] and T2* values of 35±3 ms [55] were measured on healthy volunteers while T2* values of 27±20 ms were measured on patients with myocardial fibrosis [30]. T1 values of 1100±67 ms and 950-1050 ms were measured in vivo on domestic farm pigs [56] and normal volunteers [57-59] respectively. However, no measures of MTR, ADC or FA were reported on the heart.

The relationship found between the Young’s modulus and the MRI parameters is the basis for the development of an indirect tool for the in vivo evaluation of the mechanical properties of cardiac tissues. However, these relationships vary between biological tissues and the degenerative state of the tissue. Equivalent experiments were done on intervertebral discs and showed that 45 to 80% of the Young’s modulus, the aggregate modulus, the radial permeability and the axial permeability can be explained mostly by MT and diffusion sequences [60]. On the skeletal muscle, up to 78% of the Young’s modulus can be explained by relaxation times, magnetization transfer and diffusion coefficients suggesting a linear relationship [61]. However, both studies showed changes in the relationships when the tissue is degenerated with a significant modification of the mechanical properties, suggesting that before the use of this technique to quantify the mechanical properties in vivo on patients suffering from various diseases, the relationships have to be defined for each degeneration state of the tissue that mimics the pathology.

There were some limitations to this study which warrant further investigations. The low number of tissue samples was due to the difficulty to obtain the animals’ heart within two hours of death from the slaughterhouse. Our strict observation of the 2-hour window, in contrast, permitted the uniformity of the test results however. Another limitation relates to the chamber used for the MRI acquisition, which was manufactured in acrylonitrile butadiene styrene by rapid prototyping (fused plastic deposit). The limit of this method is that small air bubbles can be trapped during the fused plastic deposit, even if high-density presets are used. Nevertheless, artifacts on the relaxation time images were removed using a filter that suppresses high values (more than 2500 ms in T1, 200 ms in T2). For the diffusion images, the use of a multi-shot echo-planar-imaging sequence decreased the distortion induced in the images by the air bubbles. Diffusion tensor imaging is often limited by a lower signal to noise ratio than in relaxation time imaging, but an estimated signal to noise ratio of 105 for our b=0 image confirms the reliability of our ADC and FA measures in the cardiac muscle tissue. From an analytical perspective, the mechanical behaviour of the cardiac muscle tissue is known to be hyperelastic [11-15] which warrant the experimental data to be fitted with a hyperelastic model instead of a linear model.

In vivo relaxometry of cardiac tissue is a well established method already used in clinical applications [25-30]. The *in-vivo* sensitivity of MTR measurements to infarct and inflammation was proved [38]. However, the application of diffusion tensor imaging of cardiac tissue *in-vivo* remains challenging, but the feasibility has already been demonstrated and should be available in the near future. Thus our method could be transferred to in an *in-vivo* study in which the mechanical properties as determined by multi-parametric MRI could be compared to the mechanical properties as determined from cine-MRI or cine-tagging-MRI associated to finite element modeling [7-11].

## Conclusions

The proposed multi-parametric MRI protocol associated to principal component analysis is a promising tool for the evaluation of mechanical properties within the left ventricular myocardium. Our *in vitro* experiments will now allow us focused *in vivo* testing on healthy and infracted hearts in order to determine useful quantitative MR-based biomarkers. Based on our data it is also possible now to perform longitudinal *in vivo* testing on ischemic and revascularized hearts. Future directions of our laboratory aim for to better understand and to implement novel MRI parameters as biomarkers of myocardial viability and potential prognostic scoring of myocardial relaxation and contraction. The next experimental step will verify the *in vivo* application of this technique: the data from an *in vivo* multi-parametric MRI acquisition on healthy and infracted pigs will be compared to the data from the same MRI protocol performed on the same animals after euthanasia, followed by cyclic mechanical tests and biochemical tests.

## Competing interests

The authors declare that they have no competing interests.

## Authors’ contributions

DP proposed the design of the study, carried out the data analysis, discussed the results and drafted the manuscript. ND participated to the results discussion and revised the manuscript. AF carried out the experiments. DC participated to the design of the study and the statistical analysis and revised the manuscript. All authors read and approved the final manuscript.

## Pre-publication history

The pre-publication history for this paper can be accessed here:

http://www.biomedcentral.com/1471-2261/13/24/prepub
